# Circadian Clock Gene Expression in the Coral *Favia fragum* over Diel and Lunar Reproductive Cycles

**DOI:** 10.1371/journal.pone.0019755

**Published:** 2011-05-06

**Authors:** Kenneth D. Hoadley, Alina M. Szmant, Sonja J. Pyott

**Affiliations:** 1 Department of Biology and Marine Biology, University of North Carolina Wilmington, Wilmington, North Carolina, United States of America; 2 Department of Biology and Marine Biology and the Center for Marine Science, University of North Carolina Wilmington, Wilmington, North Carolina, United States of America; King Abdullah University of Science and Technology, Saudi Arabia

## Abstract

Natural light cycles synchronize behavioral and physiological cycles over varying time periods in both plants and animals. Many scleractinian corals exhibit diel cycles of polyp expansion and contraction entrained by diel sunlight patterns, and monthly cycles of spawning or planulation that correspond to lunar moonlight cycles. The molecular mechanisms for regulating such cycles are poorly understood. In this study, we identified four molecular clock genes (*cry1, cry2*, *clock* and *cycle*) in the scleractinian coral, *Favia fragum,* and investigated patterns of gene expression hypothesized to be involved in the corals' diel polyp behavior and lunar reproductive cycles. Using quantitative PCR, we measured fluctuations in expression of these clock genes over both diel and monthly spawning timeframes. Additionally, we assayed gene expression and polyp expansion-contraction behavior in experimental corals in normal light:dark (control) or constant dark treatments. Well-defined and reproducible diel patterns in *cry1, cry2*, and *clock* expression were observed in both field-collected and the experimental colonies maintained under control light:dark conditions, but no pattern was observed for *cycle*. Colonies in the control light:dark treatment also displayed diel rhythms of tentacle expansion and contraction. Experimental colonies in the constant dark treatment lost diel patterns in *cry1, cry2*, and *clock* expression and displayed a diminished and less synchronous pattern of tentacle expansion and contraction. We observed no pattern in *cry1, cry2, clock*, or *cycle* expression correlated with monthly spawning events suggesting these genes are not involved in the entrainment of reproductive cycles to lunar light cycles in *F. fragum.* Our results suggest a molecular clock mechanism, potentially similar to that in described in fruit flies, exists within *F. fragum.*

## Introduction

Predictable and cyclic diel patterns of sunlight and monthly cycles of moonlight occur in most geographic locations around the globe. Accordingly, many species have evolved mechanisms to entrain behaviors to these environmental light patterns [1], [2], [3], [4], [5]. Scleractinian corals display behavioral and reproductive changes corresponding to both diel solar and monthly lunar light cycles. Many reef corals retract their tentacles during the day and extend them at night to feed [6], [7], [8], [9]. Diel light cycles may also be involved with determining the time of day of coral spawning [10]. Over longer periods of time, the lunar cycle provides a light cue that is thought to play a role in synchronization of reproductive events such as gametogenesis, spawning or larval release in some scleractinian coral species [11], [12]. Within a given geographic region, spawning time occurs simultaneously for all corals of a particular species. This precise and simultaneous release of gametes is thought to be an adaptation for increasing the probability of successful fertilization [13].

Although rhythmic coral behaviors such as diel tentacle expansion-contraction and synchronous spawning have been well characterized, little is known about the molecular signaling pathways responsible for these behaviors. In model systems such as fruit flies and mice, circadian behaviors are maintained by a well-studied core molecular “clock” composed of the transcriptional activators CLOCK and CYCLE (orthologus to BMAL1 in vertebrates) and other positive and negative regulatory components including PERIOD, TIMELESS and CRYPTOCHROME [14], [15], [16]. Molecular clock components, including the cryptochrome genes, are also thought to play fundamental roles in the timing of reproductive processes in these taxa [17].

A recent meta-analysis has shown that orthologs of many of these genes are present in the basal metazoan phylum Cnidaria, specifically, in the coral *Acropora millepora* and the sea anemone *Nematostella vectensis* [18]. Further, correlative evidence suggests that upregulation of one of the molecular clock genes, *cryptochrome* 2 (*cry2*), may play a role in the timing of spawning of the scleractinian coral *A. millepora* [19]. Whether these genes are involved in entraining cnidarian behaviors remains unclear. Based on the established roles these genes play in maintaining both shorter circadian and longer timeframe reproductive rhythms within insect and mammalian species [16], [17], [20], [21], [22], these genes may also be important in synchronizing both diel and monthly behaviors within scleractinian corals.

In this study, we investigated whether the brooding coral, *Favia fragum,* had diel or lunar cycles of *cry1, cry2, clock*, and *cycle* transcript abundance that correlated with diel sunlight cycle and/or key events in the monthly reproductive cycle of *F. fragum*. *F. fragum* is a small Caribbean reef coral that reproduces monthly throughout the year in a predictable lunar pattern [23], in contrast to *A. millepora* and many other broadcast spawning corals that reproduce annually [13], [24]. Understanding the patterns of expression of these genes will help elucidate the circadian molecular clock mechanism in corals and the evolution of clock mechanisms within the metazoan lineage.

Phylogenetic analyses of sequenced rtPCR gene products was used to confirm the presence of orthologous clock gene products within the *F. fragum* transcriptome. Quantitative PCR (qPCR) methods were then used to measure fluctuations in clock gene expression over both short term (diel) and longer term (reproductive) cycles. Behavioral outputs, specifically polyp expansion and contraction over the diel cycle, and gametogenesis over the lunar cycle were also monitored and compared to fluctuations in clock gene expression. Changes in behavior and gene expression under constant darkness were also monitored under laboratory conditions. Although we saw little evidence to support a role of clock genes in reproductive behaviors, clear fluctuations in diel expression were observed in some of the genes. These results provide further evidence to support a molecular clock mechanism similar to that within fruit flies and mice. Comparison of *F. fragum* expression patterns with other those of other cnidarian species reveals significant differences in clock gene expression profiles possibly reflecting differences in life history among species.

## Results

### Identification of Clock Genes in *Favia fragum*

**Figure 1:**
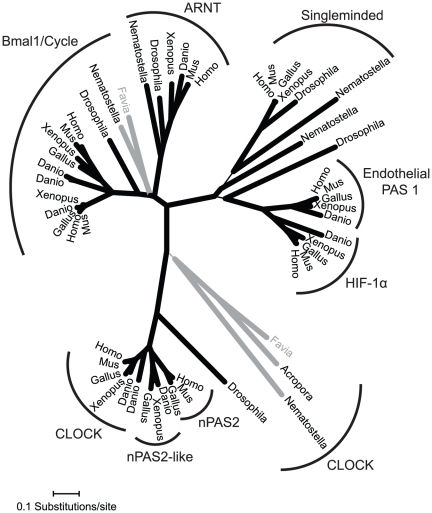
Phylogenetic relationship of the *F. fragum* genes *clock and cycle.* Tree depicts a radial consensus of 1000 bootstrap replicates using the Neighbor-Joining method with pairwise deletions and Poisson control. Analyses with 1000 bootstrap replicates under Dayhoff or JTT models were also run and resulted in similar phylogenetic relationships (data not shown). Thin lines represent nodes where less than 70% of the 1000 bootstrap replicates show support for the topology depicted above. Gray lines represent cnidarian lineages.

**Figure 2:**
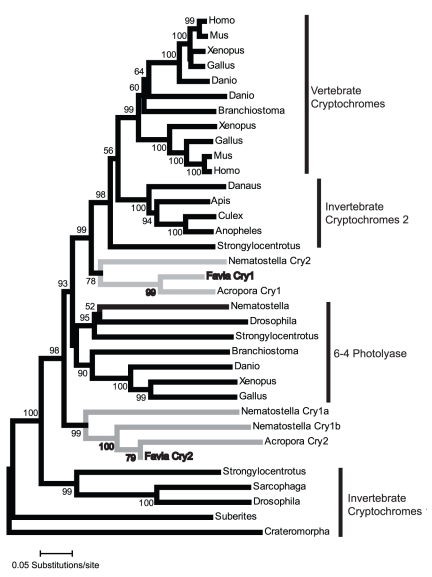
Phylogenetic relationship of *F. fragum* genes *cry1* and *cry2.* Tree depicts a consensus of 1000 bootstrap replicates using the Neighbor-Joining method with pairwise deletions and Poisson control. Analyses with 1000 bootstrap replicates under Dayhoff or JTT models were also run and resulted in similar phylogenetic relationships (data not shown). Gray lines represent cnidarian lineages.

Reverse transcription PCR with degenerate primers was used to identify and sequence partial cDNAs encoding the genes *cry1, cry2, clock*, and *cycle* within the *F. fragum* transcriptome. Gene identity was initially confirmed using BLASTn and tBLASTx searches in NCBI. Phylogenetic analyses using the neighbor joining method through MEGA 4 were also used to confirm gene identity and also identify cnidarian orthologs (Figure 1 and Figure 2; Table S1). These analyses identify inconsistency with regard to the attribution and naming of the *cry1* and *cry2* gene sequences in the two cnidarians that have been previously studied (*N. vectensis* [25] and *A. millepora* [19]), and, in this work, we use the designation originally published for *A. millepora* [19]. The resulting analyses suggest *F. fragum cry1* is most similar to *A. millepora cry1* and *N. vectensis cry2* (GenBank Accession: HQ687760), *F. fragum cry2* is most similar to *A. millepora cry2* and *N. vectensis cry1a* and *cry1b* (GenBank Accession: HQ687761), and *F. fragum clock* and *cycle* are most similar to *N. vectensis clock* (GenBank Accession: HQ687758) and *cycle* (GenBank Accession: HQ687759) respectively.

### Diel Patterns of Gene Expression

**Figure 3:**
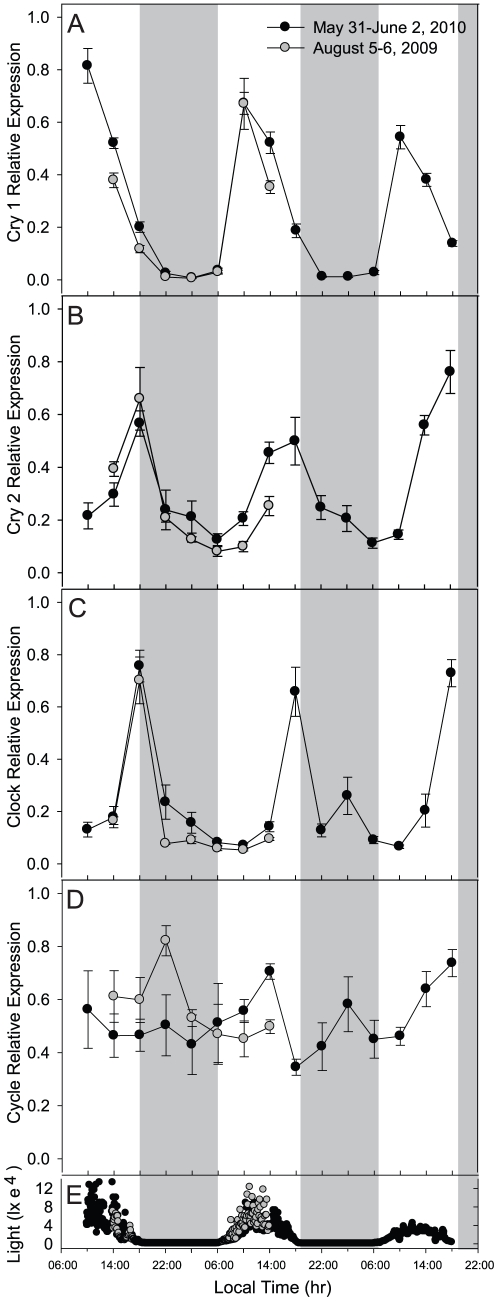
Relative expression of *F. fragum* clock genes over the diel light cycle: *F. fragum* samples were collected between August 5 and 6, 2009 (gray circles) and May 31 and June 2, 2010 (black circles). Horizontal gray bars indicate nighttime. Relative expression of *cry1* (A), cry2 (B), *clock* (C), and *cycle* (D) normalized to total RNA and *ef1*α expression (as described in the Materials and Methods) are shown as the mean ± s. e. m. & plusmns. e. m. ((((n = 5). Natural light levels (in lx) were measured every 5 min at a depth of 0.5 m (E). Dunn's method post-hoc analysis of May 2010 diel rhythms indicate the following significant (p<0.05) differences among times: c*ry1* expression at 1000 and 1400 h was significantly elevated compared to expression at 2200, 0200, and 0600 h, and expression at 1000 was also significantly elevated compared to expression at 1800 h; c*ry2* expression at 1400 and 1800 h were significantly elevated compared to expression at 0200, 0600, and 1000 h, and expression at 1800 h was also significantly elevated compared to expression at 2200 h; c*lock* expression at 1800 h was significantly elevated compared to expression at 0200, 0600, 1000, 1400, and 2200 h;c*ycle* expression showed no significant differences among sampling times (p = 0.364). Statisical analyses of the diel data collected August 2009 is provided in Table S3).

To investigate the diel expression of *cry1*, *cry2*, *clock*, and *cycle* in field-collected *F. fragum* colonies, relative transcript abundances were measured using qPCR on samples collected over a 24 h period (August 2009) and repeated over a 60 h period (May 2010). Significant diel peaks in expression were observed for *cry1* at 1000 h for both the August 2009 and May 2010 collection periods (Figure 3). Compared to expression levels measured at dawn (0600 h), dusk (1800 h), and nighttime (2200 h and 0200 h), *cry1* relative expression at (1000 h) was always elevated (p<0.05; Figure 3A). *Cry2* showed a similar pattern of cyclic expression, but with expression peaking at dusk (1800 h) and significantly elevated compared to transcript levels measured at night time (0200 h) and dawn (0600 h; p<0.05; Figure 3B). Like *cry2*, *clock* expression peaked at 1800 h (dusk) and was significantly elevated compared to levels measured at dawn (0600 h) and morning (1000 h) (p<0.05; Figure 3C). Although *cycle* showed significantly increased relative expression at night (2200 h) compared to dawn (0600 h) and midday (1000 h and 1400 h) during the August 2009 period (p<0.05; Figure 3D gray symbols), no significant diel variation was observed during the more extensive May 2010 sampling period (p = 0.129; Figure 3D black symbols).

### Light Dependence of Gene Expression and Tentacle Expansion-Contraction Behavior

To examine the light-dependence of diel patterns of gene expression, clock gene expression measured in corals maintained in the laboratory under normal light:dark conditions (control treatment) were compared to expression measured in corals maintained in constant darkness (dark treatment). Polyp expansion-contraction behaviors were also monitored during the first three days of the experiment before sampling for gene expression.

**Figure 4:**
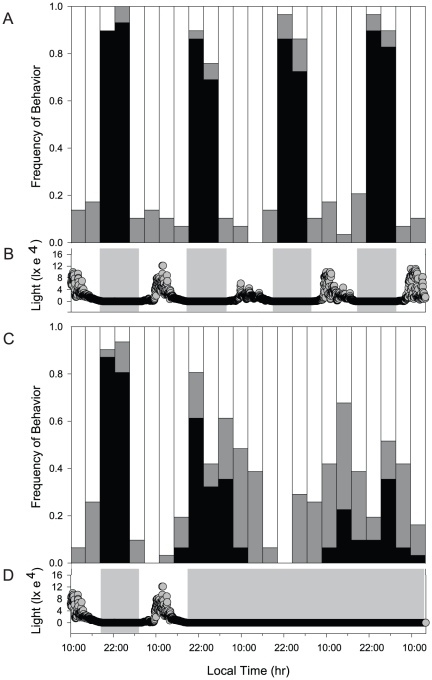
Diel polyp expansion and contraction in *F. fragum* in light:dark and constant dark treatments. The fraction of colonies with contracted (white), partially expanded (gray), or fully expanded (black) in light:dark (A; n = 29) and constant dark (C, n = 31) treatments. Light levels (lx) were measured every 5 min for light:dark (B) and constant dark treatments (D). Horizontal gray bars indicate dark periods.

During an initial 24 h normal light:dark period, all experimental corals exhibited normal polyp expansion at night and contraction during the day (Figures 4A–C). After collection of these baseline behavioral data, half of the corals remained in the normal light:dark treatment while the other half was transferred to a constant dark treatment. Given the variable levels of gene expression over the normal diel light cycle demonstrated for the field samples (Figure 3), we selected 1400 h and 0200 h of each day to perform statistical comparisons between treatments as representative of daylight and nighttime conditions.

There was no significant difference in expansion-contraction behavior between the two groups of corals at 1400 and 0200 h during the pre-treatment monitoring period (p>0.05). Corals in the normal light:dark treatment continued the expected nighttime polyp expansion and daytime contraction pattern throughout the experimental period (Figure 4A). In contrast, colonies transferred to the constant dark treatment initially showed a prolonged period of expanded and partially expanded polyps, followed by a less synchronized and longer cycle of polyp contraction and expansion (Figure 4C). Polyp expansion-contraction behaviors at 0200 and 1400 h were significantly different between treatment groups (p<0.01). These data suggest that normal polyp expansion-contraction behavior is light dependent in *F. fragum* (Figure 4A and C).

**Figure 5:**
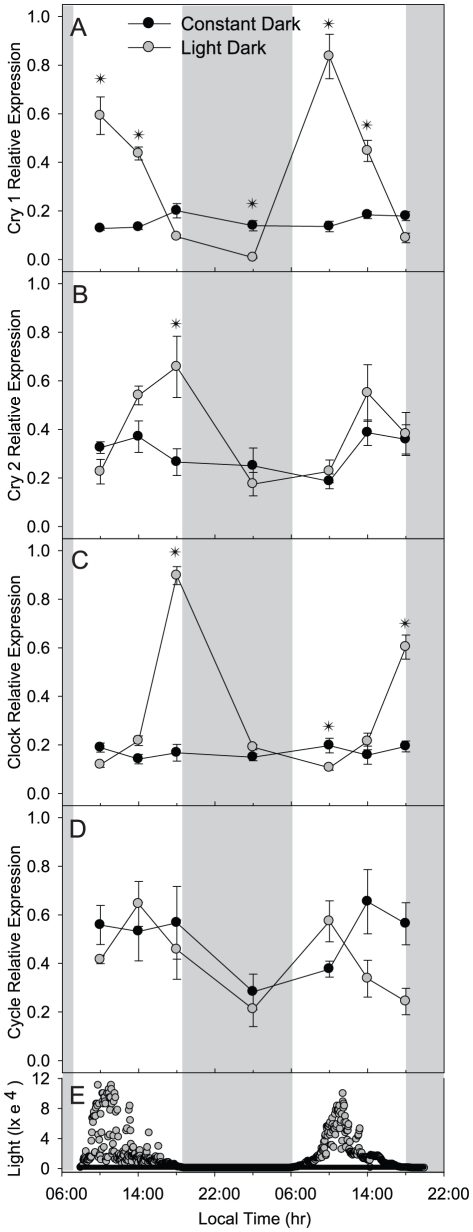
Relative expression of *F. fragum* clock genes in light:dark and constant Dark treatments. Relative expression of *cry1* (A), *cry2* (B), *clock* (C), and *cycle* (D) normalized to total RNA and *ef1*α expression (as described in the Materials and Methods) are shown as the mean ± s. e. m. (n = 4) for light:dark (white circles) and constant dark (black circles) treatments. Light levels (lx) were measured every 5 min for light:dark (E, white circles) and constant dark treatments (E, black circles). * indicates statistically significant (p<0.05) differences in gene expression between light:dark and constant dark treatments.

Relative expression of *cry1*, *cry2*, and *clock* in corals maintained in the light:dark treatment for three days prior to sampling showed peaks in transcript abundance that matched those times observed in the field (compare Figure 5A–C with Figure 3A–C): *cry1* expression was elevated at 1000 h and *cry2* and *clock* expression was elevated at 1800 h over nighttime (0200 h) and dawn (0600 h) expression levels (p<0.05). In contrast, the relative expression of *cry1, cry2*, and *clock* in corals in the constant dark treatment showed no pattern of expression over the dark sampling period (p>0.05, Figure 5A–C). Peak levels in transcript abundance for *cry1* (1000 h, for both diel cycles), *cry2* (at 1800 h for the first diel cycle), and *clock* (at 1800 h for both diel cycles) observed in corals maintained in normal light:dark were significantly elevated compared to expression at those time points in corals maintained in the dark (p<0.001). Although there was no rhythmic pattern of expression for *cry1* throughout the dark treatment, levels of expression were elevated compared to normal nighttime (0200 h) levels (p<0.001; Figure 5A). Only *cycle* showed no statistically significant difference in expression between corals maintained in normal light:dark compared to those in the dark (p = 0.52; Figure 5D). However, statistical analyses indicated that there was a small but significant increase in *cycle* expression for both light:dark and dark treatments at 1400 h compared to 0200 h (p<0.05; Figure 5D). This increase was not repeated at 1400 h on the second day and is likely due to the large, arrhythmic variation observed in *cycle* expression.

### Lunar Pattern of Expression

**Figure 6:**
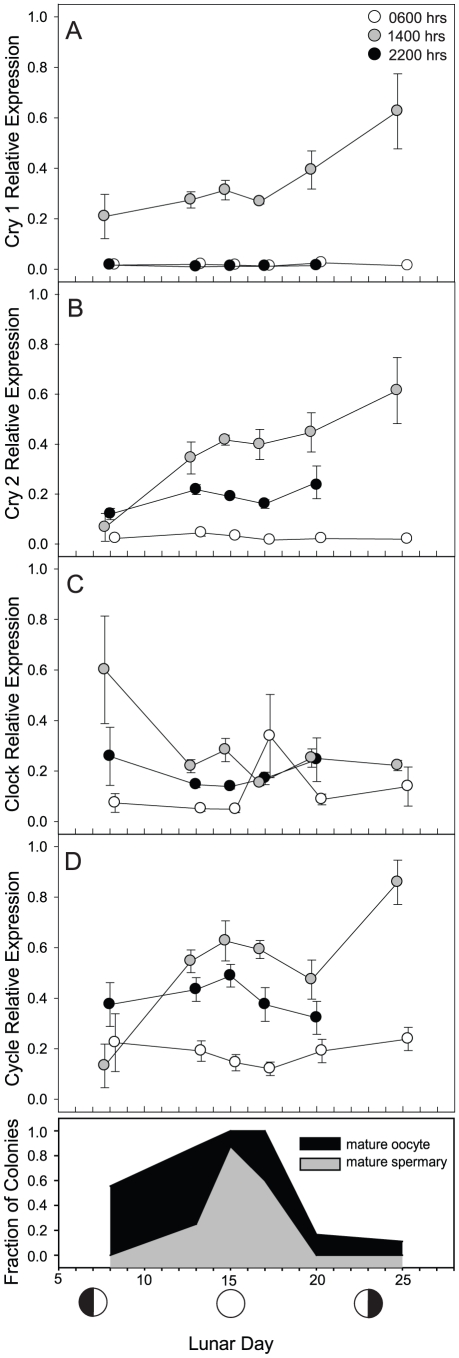
Relative expression of *F. fragum* clock genes over the lunar cycle. F. fragum samples were collected between July 7 and August 25, 2009 at 0600 h (white circles), 1400 h (gray circles) and 2200 h (black circles). Relative expression of cry1 (A), cry2 (B), clock (C), and cycle (D) normalized to total RNA and *ef1*α expression (as described in the Materials and Methods) are shown as the mean ± s. e. m. (n = 4 to 5). The fractions of F. fragum colonies determined to contain mature oocytes (black) and spermaries (gray) are shown over the same time period (E). Two-way ANOVA indicates the following significant (p<0.05) differences among times and days: cry1 expression at 1400 h was significantly elevated compared to expression at 0600 and 2200 hrs with no significant differences in expression among lunar days; cry2 expression at 1400 h was significantly elevated compared to expression at 0600 and 2200 h with expression on lunar days 20 and 25 significantly elevated compared to expression on day 8; clock expression at 1400 h and 2200 h was significantly elevated compared to expression at 0600 h with no significant differences in expression among lunar days; cycle expression at 1400 h was significantly elevated compared to expression at 0600 h and 2200 h, with expression on lunar day 25 significantly elevated compared to expression on lunar days 8, 17 and 20.

To investigate the possible involvement of *cry1, cry2, clock* and *cycle* genes in the monthly spawning cycle of *F. fragum*, relative transcript abundances were measured using qPCR in samples collected every eight hours (three samples per diel cycle) on each of five days, and two time points on a sixth day. Sampling days were strategically chosen to include specific reproductive events, especially spawning and onset of embryogenesis (days 15–20), in the *F. fragum* life cycle [23]. As expected from the diel cycle data, levels of expression for *cry1* and *cry2* were elevated in corals collected at 1400 h compared to those collected at 0600 h and 2200 h (Figure 6A, B). Expression levels of *clock*, but not *cycle*, were elevated in corals collected at 1400 and 2200 h over those at 0600 h (Figure 6C, D). Thus, for detection of lunar cycles of gene expression, comparison among lunar days was done independently for the 1400 h and 2200 h sample times.

There was a non-significant trend of increasing *cry1* transcript abundance over the sampling timeframe (Figure 6A). For *cry2,* levels of expression at 1400 h on days 20 and 25 were significantly elevated compared to transcript abundance on day 8 (p<0.01; Figure 6B). No significant difference in expression among lunar days was detected for *clock* (p = 0.369; Figure 6C). Transcript abundance of *cycle* did vary temporally, with expression levels on day 25 significantly elevated over days 8, 17 and 20 (p<0.05; Figure 6D). The biological significance, if any, of these variations is unknown since they do not correlate with any particular aspect of the reproductive cycle. Histological analysis of the colonies sampled for the lunar reproductive cycle showed that the number of colonies with mature oocytes and spermaries peaked on day 15 (the full moon) and declined to almost none on day 20 of the lunar cycle, indicating that spawning had taken place during the sampling interval (Figure 4E). Thus, the *F. fragum* samples collected in this study were undergoing the same pattern of gamete maturation and spawning over the collection time period as described previously [23].

## Discussion

In this work we verified the expression of the core clock genes *cry1*, *cry2*, *clock*, and *cycle* in the brooding coral *F. fragum*. Through a variety of field and laboratory investigations, we characterized the light-dependence of gene expression and also correlated gene expression to diel tentacle expansion-contraction and lunar reproductive events. Significant and reproducible diel cycles in *cry1*, *cry2*, and *clock* expression were observed in field-collected *F. fragum* over several sampling periods. Specifically, *cry1* showed elevated expression at 1000 h and both *cry2* and *clock* showed elevated expression at 1800 h. Both genes had minimal levels of expression in nighttime samples. In contrast, *cycle* expression was highly variable with no discernable pattern of expression. These findings are consistent with observations of *cryptochrome* expression in *A. millepora* [19] and largely consistent with observations of *cryptochrome*, *clock*, and *cycle* expression in *N. vectensis* [25]. In *A. millepora,* both *cry1* and *cry2* (similar to *F. fragum cry1* and *cry2*) had significantly elevated expression in response to light [19]. In *N. vectensis cry1a* and *cry1b* (similar to *F. fragum cry2*) and *clock* showed elevated expression in response to light, with *cry1a* and *cry1b* peaking during the middle of the light period and *clock* peaking in expression just before the onset of the dark period [25]. Also similar to our observations in *F. fragum*, in *N. vectensis* there was no light-dependent variation in *cycle* expression.

To more carefully characterize the light dependence of clock gene expression in *F. fragum* and also correlate expression to a well-documented coral diel behavior, tentacle expansion and contraction [6], [7], [8], [9], we examined colonies of *F. fragum* in the laboratory under controlled light:dark and constant dark conditions. Corals showed diel peaks in expression of *cry1*, *cry2*, and *clock* that were dependent on light and not observed under conditions of constant dark. These results are consistent with observations of the loss of diurnal peaks in expression of *cry1* and *cry2* in *A. millepora* [19] and *cry1a* and *cry1b* (similar to *F. fragum cry2*) in *N. vectensis* in prolonged darkness. We observed a diminished rhythmicity in tentacle expansion and contraction when colonies of *F. fragum* were kept in constant dark. This loss in behavioral rhythmicity was correlated to a complete loss in peaks of *cry1*, *cry2*, and *clock* expression under constant dark. Correlation of diel behaviors with clock gene expression have not been previously examined in cnidarians. Although our data are only correlative, they suggest further examination of the mechanism by which the genes comprising the molecular circadian clock in *F. fragum* and other cnidarians may regulate diel behaviors like tentacle expansion and retraction.

Although there are similarities between the patterns we found in *F. fragum* and those reported for *N. vectensis* that suggest largely similar mechanisms of circadian clock function in these two cnidarians, we did observe differences in *cryptochrome* gene expression. Specifically, while both *cry1* and *cry2* in *F. fragum* were significantly elevated during the day, expression of *N. vectensis cry2* (similar to *F. fragum cry1*) remained relatively stable over the experimental light and dark periods [25]. Differences in *cryptochrome* gene expression may reflect differences in the life history strategies of *F. fragum* and *N. vectensis*. *N. vectensis* does not have symbiotic algae nor does it secrete a carbonate skeleton. Both feeding and calcification have diel cycles in many scleractinian corals and could require different or additional molecular mechanisms to coordinate those processes. Perhaps differences in *cry1* and *cry2* gene expression relative to *clock* gene expression in *F. fragum* reflect these daytime calcification and symbiosis [26] and nocturnal feeding [7], [12] behaviors. Indeed, diurnal and nocturnal animals are known to show differences in the relative timing of *cry* and *clock* gene expression [27], [28]. Differences in expression patterns of the clock genes in cnidarians may also regulate daytime and nighttime behaviors.

The diminished rhythmicity of *F. fragum* tentacle expansion-contraction and clock gene expression in the absence of light contrasts observations in the fruit fly and many vertebrates, which continue to display rhythms in levels of activity and sleep cycles, albeit sometimes with changes in period length [16], [29], [30], [31], [32]. Interestingly, fruit fly and mouse knockouts of the clock gene *period* display diel clock gene expression under natural light cycles and arrhythmic clock gene expression under constant dark conditions [33], [34], [35]. We were unable to isolate *period* within the *F. fragum* transcriptome. Analyses of the fully sequenced *N. vectensis* genome also failed to identify *period* and has led to the suggestion that this gene may have been lost within the cnidarians or evolved after the split between cnidarians and bilaterian metazoans [25]. Because PERIOD is an essential regulator of the molecular clock in the fruit fly and mouse [15], [16], [36], its absence in cnidarians suggests differences between cnidarians and bilaterians in the negative regulatory loop of the molecular clock involved in the preservation of biological rhythms in the absence of light.

In addition to examining the diel expression of clock genes in *F. fragum*, we also examined the expression of these genes over the lunar reproductive cycle. We found no evidence to support a correlation between increased expression of several clock genes and timing of spawning by *F. fragum.* Histological analyses of gametogenesis in the colonies of *F. fragum* used to quantify *cry1*, *cry2*, *clock*, and *cycle* expression during a three-week sampling period centered on the full moon showed that, although spawning occurred as expected just after the full moon, it was not associated with a relative peak in expression of any of the clock genes. There was, however, a gradual and significant increase in expression of *cry2* and *cycle* 10 days after the full moon that does not appear to be related to the reproductive cycle. These results contrast with observations of increased *cry2* expression in *A. millepora* (Family Acroporidae) at 0000 h on the full moon night of spawning compared to that of non-spawning full moon or new moon nights [19]. Our lunar sampling time points (at 0600 h, 1400 h, and 2200 h) were chosen to detect peaks in *cry2* expression expected to occur at 1800 h, based on our diel sampling, or at 0000 h, based on these earlier observations in *A. millepora* [19]. Nonetheless, it is possible that we failed to detect transient peaks in *cry2* expression that occurred between our lunar sampling time points. Our available data nevertheless do not suggest an involvement of *cry2* in regulating coral spawning in *F. fragum* (Family Faviidae), suggesting reanalysis of these earlier findings [19] and/or different molecular mechanisms regulating spawning in different families of scleractinian corals (and specifically Acroporid and Faviid families).

The clear diel fluctuations in *cry1, cry2* and *clock* gene expression confirm a light-dependent, molecular clock mechanism exists within the scleractinian coral *F. fragum* and is shared with other cnidarians, including *A. millepora* [19] and *N. vectensis* [25]. Corals with photosynthetic endosymbionts, like *F. fragum* and *A. millepora*, also undergo diel cycles of hyperoxia (by day) and hypoxia (by night) [37]. In A. millepora, glycolytic genes, potentially under regulation of an ortholog of the hypoxia-inducible factor (HIF) 1α transcription factor, show diel peaks in expression at night that disappear in conditions of constant dark [38]. These findings suggest the existence of a parallel molecular mechanism that allows corals to tune their circadian behaviors to the internal environment established by their endosymbionts. Evidence for such a clock in *F. fragum* but not in the non-symbiotic *N. vectensis*, would provide further support for the life history specificity of this mechanism.

In conclusion, the diel fluctuation of *clock* and not *cycle* and also the loss of diel fluctuation of *cry1*, *cry2*, and *clock* in conditions of constant dark provide additional support for an ancestral origin of the molecular circadian clock in cnidarians [25]. Firstly, although *clock* and *cycle* are the essential positive transcriptional regulators of the molecular clock in the fruit fly and mouse, diel expression of these genes differs between these groups [15], [16]. Specifically, *clock* but not *cycle* shows diel variations in expression in the fruit fly, whereas *bmal1* (the mammalian ortholog of *cycle*) but not *clock* shows diel variation in mouse [15]. The cyclical expression of *clock* but not *cycle* within *F.* fragum, therefore, suggests a molecular circadian clock mechanistically more similar to that found in the fruit fly. Secondly, the arrhythmic expression of *clock*, *cry1* and *cry2* in conditions of constant dark and the absence of *period* in cnidarians examined to date [25] suggest an ancestrial mechanism of circadian regulation in cnidarians that does not involve the *period* gene. Future research on the cnidarian molecular clock should focus on the protein interactions within this system and how they produce the novel clock gene expression profiles observed in this work.

## Materials and Methods

### Study Site

All field and laboratory experiments were performed with permissions obtained from the Department of Marine Science at the University of Puerto Rico Mayaguez. Colonies of *F. fragum* were collected along the western side of Isla Magueyes, Puerto Rico, during August 2009 and June 2010. Only healthy colonies visually assessed to be free of disease, with full tissue coverage and more than 20 polyps were selected for use in this study. Coral samples collected as described below were shipped to UNC Wilmington on either dry ice or in liquid nitrogen for RNA extraction, or in 70% ethanol for histology. Water temperature and light intensity at the collection site were recorded using Hobo data loggers (Onset Computer Corporation, Pocasset, Massachusetts) throughout each collection period. According to recorded sunrise/sunset and photoperiod data for Puerto Rico, the total difference in photoperiod between the first and last day of our sampling is less than 15 min. Light intensity data recorded at our site confirms little change in sunrise/sunset and photoperiod.

### Sample Collections

Diel Sampling: On 5 August 2009, the day of the full moon, five colonies were collected every 4 h over a 24 h period at 1400, 1800, 2200, 0200, 0600, 1000, and 1400 h local time. A second series of samples were collected every 4 h over a 60 h period starting on 31 May 2010 at 1000 h local time, spanning days 19 through 21 of that lunar month.

Lunar cycle: Sample dates were 29 July and 3, 5, 7, 10, and 15 August 2009, which encompass days 7 through 25 of the lunar cycle. On each sampling date, five colonies were collected every 8 h at 0600, 1400, and 2200 local time for a total of 15 total collected colonies.

Coral colonies were cleaved in half. One half was immediately flash frozen in liquid nitrogen for future RNA extraction. The other half was prepared for histology by fixing in Zenker's formaldehyde [23] for 5 h, decalcifying in 10% HCl for approximately 24 to 48 h, and storing the tissue sample in 70% ethanol for transportation back to UNC Wilmington.

### Light Manipulation Experiments

During May 2010, 60 freshly collected colonies were transferred to an outdoor, flow-through water table measuring 1.83 m long by 0.6 m wide with a flow rate of 0.4 liters s^−1^, under full natural light. Seawater for the Magueyes seawater system is pumped from a location near the sampling site and, therefore, is similar to the water quality and temperature of field-collected corals. Monitoring of polyp expansion began 4 h after initial collection and was monitored thereafter every 4 h for 5 days. Colonies were visually scored as having fully expanded polyps (1), partially expanded polyps (0.5), or retracted polyps (0) as described previously [6]. After a 24 h acclimation period, 30 of the corals were moved at night (in the dark) to an adjacent water table covered with a sheet of dark plastic to provide constant dark. Polyp expansion was monitored in the constant dark treatment with a low intensity red light emitting diode (LED) head lamp. After 72 h, five colonies were collected from each treatment, and immediately flash frozen in liquid nitrogen for RNA extraction. Additional sets of colonies were collected over a 2 day period at 1000, 1400, 1800, 0200, 1000, 1400 and 1800 local time. Throughout the experiment, water temperature and light intensity in both tanks were recorded using Hobo data loggers (Onset Computer Corporation).

### RNA Extraction

Frozen coral samples were ground into a powder using a mortar and pestle chilled on a bed of dry ice. Total RNA was extracted and purified from each sample using TRIzol Reagent (Invitrogen) and Pure Link (Invitrogen) clean up kits and a modified version of the manufacturer's protocols that included an additional chloroform extraction and sodium acetate precipitation step. Purified RNA samples were then analyzed using a NanoDrop 2000 spectrophotometer (ThermoScientific) and a 2100 Bioanalyzer (Agilent Technologies) to assess RNA quantity and quality. RNA integrity numbers were on average 9.2 and always >8.4. Only samples with concentrations greater than 50 ng µl^−1^ were used for subsequent analyses.

### Reverse Transcription PCR Amplification (rtPCR)

Reverse transcription PCR reactions were performed using 200 U µl^−1^ Superscript III reverse transcriptase (Invitrogen) and oligo (dT) primers following the manufacturer's protocols. cDNA reactions for rtPCR and also quantitative real time PCR were made from 800 ng and 10 ng of total RNA respectively. For rtPCR amplification, 1 µL of cDNA was used in each 50 µL reaction. Degenerate primers for *cry 1*, *cry 2*, *clock*, *cycle* and the housekeeping gene *ef1*α (*elongation factor 1*α) were designed using Primer Express software (AppliedBiosystems) or Primer Quest software (Integrated DNA Technologies) and available sequence data for *N. vectensis* and *A. millepora* (Table S2). rtPCR reactions were carried out with either TSG polymerase (Bio-Basics, Inc.) for *cry1* and *cry2* or high fidelity polymerase (BioRad) for *clock* and *cycle* and then purified using QIAquick PCR purification kits (Qiagen). For rtPCR reactions using TSG polymerase, each 50 µL reaction contained 0.4 µL polymerase, 1 µL 10 mM dNTPs, 5 µL 10X buffer, 3.5 µL 25 mM MgSO_4,_ 1 µL each of 50 mM forward and reverse primer, 36.1 µL ddH_2_O (nuclease-free water, GrowCells) and 2 µL of cDNA. All rtPCR reactions using TSG polymerase were performed using the following protocol: 94°C for 1∶20 min followed by 30–40 cycles of 94°C for 40 s, 50–60°C for 40 s, 70°C for 1 min and a final extension at 72°C for 9 min. For rtPCR reactions using high fidelity polymerase, each 50 µL reaction contained 0.5 µL polymerase, 1 µL 10 mM dNTPs, 10 µL 5X buffer, 1 µL 25 mM MgCL_2,_ 1 µL each of 50 mM forward and reverse primer, 34.5 µL ddH_2_O (nuclease-free water, GrowCells) and 1 µL of cDNA. All rtPCR reactions using high fidelity polymerase were performed using the following protocol: 98°C for 30 s followed by 30–40 cycles of 98°C for 8 s, 50–65°C for 25 s, 72°C for 1 min and a final extension at 72°C for 8 min. Purified PCR products were sequenced commercially (MacrogenUSA, Maryland). Resulting sequences were assembled using Sequencher software (Gene Codes Corporation) and sequence identity was confirmed using BLASTx and tBLASTx searches through the NCBI server on the Gen Bank database.

### Quantitative Real Time PCR Assay (qPCR)

Primers for qPCR were designed with Primer-Quest software (Integrated DNA Technologies) or PrimerExpress (Applied Biosystems; Table 1). qPCR reactions were performed using a Bio-Rad iQ5 real time detection system with 2X SYBR Green master mix (BioRad). Each 25 µL reaction contained 12 µL SYBR Green, 0.1 µL 25 mM MgCl_2,_ 0.25 µL each of 10 mM forward and reverse primer, 9.9 µL ddH_2_O (nuclease-free water, GrowCells) and 2 µL of cDNA. All qPCR reactions were performed using the following thermal profile: 50°C for 2 min, 95°C for 10 min followed by 45 cycles of 95°C for 15 s, 59°C for 1 min. Samples were run in 96 well plates with optical film. Standards were constructed from pooled total RNA samples from multiple time points and diluted in a 4-fold dilutions series prior to cDNA synthesis. Standards were run in triplicate and samples run in duplicate. Immediately after the qPCR reaction, a dissociation curve between 59 and 95°C in 0.5°C intervals was performed. Efficiency values for each gene were calculated using the formula *E* = 10^(−1/slope)^ [39]. Average efficiency values for each gene are reported along with primer sets (Table 1). Correlation coefficients (R^2^) for all genes were between 0.975 and 1.00. All data were normalized to *elongation factor 1 alpha* (GenBank Accession: HQ687762) expression from the same sample and presented as a ratio relative to *ef1*α. The gene *ef1*α was used to normalize data because it showed no significant variation throughout our sampling series. Relative expression values for each gene were calculated by dividing the above value by the highest value within each gene assay. Negative control reactions were carried out on a subset of the samples and on average contributed less than 0.1% of the overall signal. To exclude the possibility of amplification of cDNA from symbiotic algae (zooxanthellae) in the corals, all qPCR primers were screened by rtPCR against cDNA and genomic DNA extracted from *Symbiodinium sp* to ensure no amplification (algal samples and DNA provided by Dr. Mary Alice Coffroth, State University of New York, Buffalo).

**Table 1:** qPCR primers.

Gene	Forward Primer (5′-3′)	Reverse Primer (5′-3′)	Avg. Efficiency (%)	Size (bp)
*ef1*α	ATCAGGTGATGCTGCCATTGTCTC	GCCAACAGCTACAGTCTGCTTCAT	101.5	124
*cry1*	CAGGAAATCCTTTCAGGACTGGCA	ATGCTGGTAACTGGATGTGGCTCT	97.1	143
*cry2*	TTGCGGATAGAGGGTTGGATTCCT	CCACAGCCAGTTGCCTACATTCAA	101.8	158
*clock*	CGACTACTGTGGTCCTGAAGATGTC	CGCAACCAAACCCAAGATTGT	100.1	125
*cycle*	TGTAATGCCATGTCACGCAAGCTG	AGCCATCAGCTGCCTCAAGAATCA	98.8	169

### Phylogenetic Analysis of Clock and Cryptochrome Genes

A translated sequence for *F. fragum cycle* (GenBank Accession: HQ687759) was queried under BLASTp in NCBI for the top 500 sequence hits. Similarly, the translated sequence for *F. fragum cry1* (GenBank Accession: HQ687760) was also queried using BLASTp in NCBI for the top 250 sequence hits. Both *clock* and *cry1* results were then reduced to a total of 54 and 37 sequences respectively to reflect a diverse range of taxa and genes identified from the BLASTp query. For the *clock* alignment, translated sequences for *A. millepora* (gi|222781555, gi|222807278), *F. fragum clock* (GenBank Accession: HQ687758) and *N. vectensis* (gi|156359347, gi|156373864, gi|156402728, gi|156397887, gi|156392022) were also included. For the *cryptochrome* alignment, translated sequences for *F. fragum cry2* (GenBank Accession: HQ687761), *A. millepora* (gi|145881071, gi|145881069) and *N. vectensis* (gi|156353900, gi|156383457, gi|156378195, gi|156383455) were added. Both sets were aligned using clustalx 2.0.11 software. Phylogenetic analyses were then performed using the Neighbor-Joining method with pair-wise deletions in MEGA 4. Sequences were analyzed with all four models, p-distance, Poisson correction, Dayhoff and JJT (1000 bootstrap replicates).

### Assessment of Reproductive Stage

To confirm that the *F. fragum* colonies sampled during the lunar sampling schedule were undergoing the expected monthly pattern of reproduction, histology was used to detect the presence of mature oocytes and spermaries in a subset of the samples analyzed by qPCR. Decalcified coral tissues were processed and stained using methods described previously [23]. Briefly, samples were dehydrated in ethanol, cleared with xylene and embedded in Paraplast-Plus. 7 µm thick sections were stained with Heidenhain's azocarmine-aniline blue. The presence of mature spermaries and oocytes within each colony was determined by examining 4 to 8 polyps per colony. The data are presented as the proportion of colonies on any given sampling day with mature gametes (n = 5 to 12 colonies). Mature oocytes were identified by their large size (>250 µm), distinctive indentation of the nuclei, and dark red coloration. Mature spermaries were identified by the distinctive bouquets of tails.

### Statistical Analyses

Proportional qPCR data for all sampling sets were either log arcsine or log10 transformed prior to statistical analysis and analyzed using SigmaPlot 11 (Systat Software Inc). Data from the diel sampling set from 2009 and 2010 were analyzed separately using a Kruskal-Wallis one-way Analysis on Ranks with a Dunn's post-hoc test with the factor being time. Data points from the same time for different days were not found to be statistically significantly different and were, therefore, pooled and analyzed over a single 24 h time period. Gene expression data for the light manipulation experiment and for samples collected over the lunar cycle were analyzed using a two-way ANOVA with a Holm-Sidak post-hoc test (time of day and lunar day as the two factors for the lunar set, and time of day and treatment for the light manipulation set). Analysis of interactions within the lunar cycle was not possible because of an incomplete data set (2200 h day 25 missing). The data for *cry1, cry2* and *clock* expression over the lunar cycle failed the normality test. However, a normal probability plot of each assay suggested that failure to meet normality was only attributed to one or two data points. For polyp expansion-contraction behavior, we were interested in identifying differences between treatments during the daytime and night time on each day of the experiment. We performed separate contingency table analysis to compare behavior between treatments at 0200 h and 1400 h of each day. These times were specifically chosen because they correspond to times associated with maximal expected tentacle expansion (0200 h) and contraction (1400 h) based on baseline data.

## Supporting Information

Table S1 **GI numbers for sequences used in phylogenetic analyses.**
(DOCX)

Table S2 **Degenerate primer sets.** Degenerate primers were based on alignments using *A. millepora* and *N. vectensis* sequences using Sequencher software. Primers were selected using PrimerExpress software.
(DOCX)

Table S3 **Statistical analyses of August 2009 diel expression data.** Groups showing statistically significant (p<0.05) differences in gene expression as determined using a Kruskal-Wallis one-way analysis on ranks with a Dunn's method post-hoc.
(DOCX)

## References

[1] Challet E (2010). Interactions between light, mealtime and calorie restriction to control daily timing in mammals.. Journal of Comparative Physiology B-Biochemical Systemic and Environmental Physiology.

[2] Challet E, Caldelas I, Graff C, Pevet P (2003). Synchronization of the molecular clockwork by light- and food-related cues in mammals.. Biological Chemistry.

[3] Xu Y, Mori T, Johnson CH (2003). Cyanobacterial circadian clockwork: roles of KaiA, KaiB and the kaiBC promoter in regulating KaiC.. Embo Journal.

[4] Panda S, Hogenesch JB, Kay SA (2002). Circadian rhythms from flies to human.. Nature.

[5] Vollmers C, Gill S, DiTacchio L, Pulivarthy SR, Le HD (2009). Time of feeding and the intrinsic circadian clock drive rhythms in hepatic gene expression.. Proceedings of the National Academy of Sciences of the United States of America.

[6] Sweeney BM (1976). Circadian rhythms in corals, particularly Fungiidae.. Biological Bulletin.

[7] Yonge CM (1940). The biology of reef-building corals.. Great Barrier Reef Expedition (1928-1929):.

[8] Sebens KP, Deriemer K (1977). Diel cycles of expansion and contraction in coral reef anthozoans.. Marine Biology.

[9] Lasker HR (1979). Light dependent activity patterns among reef corals: *Montastrea cavernosa*.. Biological Bulletin.

[10] Brady AK, Hilton JD, Vize PD (2009). Coral spawn timing is a direct response to solar light cycles and is not an entrained circadian response.. Coral Reefs.

[11] Jokiel PL, Ito RY, Liu PM (1985). Night irradiance and synchronizationof lunar release of planula larvae in the reef coral *Pocillopora damicornis*.. Marine Biology.

[12] Abe V (1939). On the expansion and contraction of the polyp of a reef coral *Caulastrea furcata* Dana.. Palao Tropical Biological Station Studies.

[13] Baird AH, Guest JR, Willis BL (2009). Systematic and biogeographical patterns in the reproductive biology of scleractinian corals.. Annual Review of Ecology Evolution and Systematics.

[14] Lin CT, Todo T (2005). The cryptochromes.. Genome Biology.

[15] Looby P, Loudon ASI (2005). Gene duplication and complex circadian clocks in mammals.. Trends in Genetics.

[16] Hardin PE (2009). Molecular mechanisms of circadian timekeeping in *Drosophila*.. Sleep and Biological Rhythms.

[17] Dolatshad H, Davis FC, Johnson MH (2009). Circadian clock genes in reproductive tissues and the developing conceptus.. Reproduction Fertility and Development.

[18] Vize PD (2009). Transcriptome analysis of the circadian regulatory network in the coral *Acropora millepora*.. Biological Bulletin.

[19] Levy O, Appelbaum L, Leggat W, Gothlif Y, Hayward DC (2007). Light-responsive cryptochromes from a simple multicellular animal, the coral *Acropora millepora*.. Science.

[20] Dolatshad H, Campbell EA, O'Hara L, Maywood ES, Hastings MH (2006). Developmental and reproductive performance in circadian mutant mice.. Human Reproduction.

[21] Boden MJ, Kennaway DJ (2006). Circadian rhythms and reproduction.. Reproduction.

[22] Sandrelli F, Costa R, Kyriacou CP, Rosato E (2008). Comparative analysis of circadian clock genes in insects.. Insect Molecular Biology.

[23] Szmant-Froelich A, Reutter M, Riggs L (1985). Sexual reproduction of *Favia fragum* (Esper): lunar patterns of gametogenesis, embryogenesis and planulation in Puerto-Rico.. Bulletin of Marine Science.

[24] Harrison PL, Babcock RC, Bull GD, Oliver JK, Wallace CC (1984). Mass spawning in tropical reef corals.. Science.

[25] Reitzel AM, Behrendt L, Tarrant AM (2010). Light entrained rhythmic gene expression in the sea anemone *Nematostella vectensis*: the evolution of the animal circadian clock.. PLoS One.

[26] Kawaguti S, Sakaumoto D (1948). Bull Oceanogr Inst Taiwan.

[27] Kume K, Zylka MJ, Sriram S, Shearman LP, Weaver DR (1999). mCRY1 and mCRY2 are essential components of the negative limb of the circadian clock feedback loop.. Cell.

[28] Caldelas I, Poirel VJ, Sicard B, Pevet P, Challet E (2003). Circadian profile and photic regulation of clock genes in the suprachiasmatic nucleus of a diurnal mammal Arvicanthis ansorgei.. Neuroscience.

[29] Thresher RJ, Vitaterna MH, Miyamoto Y, Kazantsev A, Hsu DS (1998). Role of mouse cryptochrome blue-light photoreceptor in circadian photoresponses.. Science.

[30] Chong NW, Chaurasia SS, Haque R, Klein DC, Iuvone PM (2003). Temporal-spatial characterization of chicken clock genes: circadian expression in retina, pineal gland, and peripheral tissues.. Journal of Neurochemistry.

[31] Honma S, Ikeda M, Abe H, Tanahashi Y, Namihira M (1998). Circadian oscillation of BMAL1, a partner of a mammalian clock gene clock, in rat suprachiasmatic nucleus.. Biochemical and Biophysical Research Communications.

[32] Zhu HS, Green CB (2001). Three cryptochromes are rhythmically expressed in *Xenopus laevis* retinal photoreceptors.. Molecular Vision.

[33] Zheng BH, Albrecht U, Kaasik K, Sage M, Lu WQ (2001). Nonredundant roles of the mPer1 and mPer2 genes in the mammalian circadian clock.. Cell.

[34] Hardin PE, Hall JC, Rosbash M (1992). Behavioral and molecular analyses suggest that circadian output is disrupted by disconnected mutants in *D. melanogaster*.. EMBO Journal.

[35] Hardin PE, Hall JC, Rosbash M (1992). Circadian oscillations in period gene messenger RNA levels are transcriptionally regulated.. Proceedings of the National Academy of Sciences.

[36] Ko CH, Takahashi JS (2006). Molecular components of the mammalian circadian clock.. Human Molecular Genetics.

[37] Kuhl M, Cohen Y, Dalsgaard T, Jorgensen BB, Revsbech NR (1995). Microenvironment and photosynthesis of zooxanthellae in scleractinian corals studied with microsensors for 02p, H and light.. Mar Ecol Prog Ser.

[38] Levy O, Kaniewska P, Alon S, Eisenberg E, Karako-Lampert S (2011). Complex diel cycles of gene expression in coral-algal symbiosis.. Science.

[39] Rasmussen R, Meuer S, Wittwer C, Nakagawara K (2001). Quantification on the LightCycler.. Rapid Cycle Real-time PCR, Methods and Applications:.

