# Supplementary material for: Circadian Clock Gene Expression in the Coral Favia fragum over Diel and Lunar Reproductive Cycles
Source: PLoS One. 2011 May 6;6(5):e19755. doi: 10.1371/journal.pone.0019755 (PMC3089635; doi:10.1371/journal.pone.0019755)
Supplement: Table S1 — GI numbers for sequences used in phylogenetic analyses. (DOCX) [file pone.0019755.s001.docx]

| **Gene** | **Species** | **NCBI Protein GI** |
| --- | --- | --- |
| *Vertebrate cryptochromes* | *Homo sapiens* | 4758072 |
|  | *Mus musculus* | 6681031 |
|  | *Xenopus tropicalis* | 291290511 |
|  | *Gallus gallus* | 45383636 |
|  | *Danio rerio* | 55251266 |
|  | *Danio rerio* | 66910245 |
|  | *Branchiostoma floridae* | 260829102 |
|  | *Xenopus laevis* | 147901075 |
|  | *Gallus gallus* | 45383642 |
|  | *Mus musculus* | 27312016 |
|  | *Homo sapiens* | 3327130 |
| *Invertebrate cry2* | *Danaus plexippus* | 77166866 |
|  | *Culex quinquefasciatus* | 170059953 |
|  | *Anopheles gambiae* | 78191297 |
|  | *Apis mellifera* | 136255185 |
|  | *Strongylocentrotus purpuratus* | 115738187 |
|  | *Nematostella vectensis* | 156353900 |
|  | *Acropora millepora* | 145881071 |
|  | *Favia fragum* | HQ687760 |
| *6-4 Photolyase* | *Strongylocentrotus purpuratus* | 72012168 |
|  | *Drosophila melanogaster* | 3986298 |
|  | *Branchiostoma floridae* | 260800305 |
|  | *Nematostella vectensis* | 156383457 |
|  | *Danio rerio* | 27882257 |
|  | *Xenopus tropicalis* | 194332801 |
|  | *Gallus gallus* | 110626125 |
| *Invertebrate cry1* | *Favia fragum* | HQ687761 |
|  | *Acropora millepora* | 145881069 |
|  | *Nematostella vectensis* | 156378195 |
|  | *Nematostella vectensis* | 156383455 |
|  | *Strongylocentrotus purpuratus* | 72115950 |
|  | *Drosophila melanogaster* | 17137248 |
|  | *Sarcophaga bullata* | 210136298 |
|  | *Suberites domuncula* | 293321543 |
|  | *Crateromorpha meyeri* | 293321545 |
| *Cycle/bmal1* | *Homo sapiens* | 2094735 |
|  | *Mus musculus* | 34098759 |
|  | *Gallus gallus* | 47825375 |
|  | *Xenopus tropicalis* | 291290509 |
|  | *Danio rerio* | 30231256 |
|  | *Danio rerio* | 28279879 |
|  | *Danio rerio* | 94733888 |
|  | *Danio rerio* | 169158685 |
|  | *Xenopus tropicalis* | 156717516 |
|  | *Gallus gallus* | 45383840 |
|  | *Mus musculus* | 187466535 |
|  | *Homo sapiens* | 17979654 |
|  | *Drosophila melanogaster* | 24667005 |
|  | *Nematostella vectensis* | 156359347 |
|  | *Favia fragum* | HQ687759 |
| *ARNT* | *Nematostella vectensis* | 156373864 |
|  | *Drosophila melanogaster* | 13124680 |
|  | *Xenopus laevis* | 147903135 |
|  | *Danio rerio* | 113674860 |
|  | *Mus musculus* | 74220117 |
|  | *Homo sapiens* | 30795242 |
| *clock* | *Favia fragum* | HQ687758 |
|  | *Acropora millepora* | 222781555 |
|  | *Nematostella vectensis* | 156402728 |
|  | *Drosophila melanogaster* | 3219726 |
|  | *Danio rerio* | 55962414 |
|  | *Danio rerio* | 190338076 |
|  | *Xenopus tropicalis* | 6466210 |
|  | *Gallus gallus* | 4903294 |
|  | *Mus musculus* | 148705948 |
|  | *Homo sapiens* | 6478192 |
| *nPAS* | *Homo sapiens* | 118572678 |
|  | *Mus musculus* | 80479005 |
|  | *Gallus gallus* | 71896369 |
| *nPAS2-like* | *Gallus gallus* | 118089716 |
|  | *Xenopus tropicalis* | 301610093 |
|  | *Danio rerio* | 30231248 |
| *singleminded* | *Homo sapiens* | 194239684 |
|  | *Mus musculus* | 1213283 |
|  | *Xenopus tropicalis* | 301624571 |
|  | *Gallus gallus* | 118083884 |
|  | *Drosophila melanogaster* | 3769480 |
|  | *Nematostella vectensis* | 156397887 |
|  | *Nematostella vectensis* | 156392022 |
|  | *Drosophila melanogaster* | 24651293 |
| *Endothelial PAS1* | *Homo sapiens* | 40254439 |
|  | *Mus musculus* | 2895756 |
|  | *Gallus gallus* | 46048879 |
|  | *Xenopus tropicalis* | 148227427 |
|  | *Danio rerio* | 292619504 |
| *HIF1α* | *Danio rerio* | 125833414 |
|  | *Xenopus tropicalis* | 102621855 |
|  | *Gallus gallus* | 45383550 |
|  | *Mus musculus* | 148704554 |
|  | *Homo sapiens* | 3790535 |
