# Supplementary material for: Circadian Clock Gene Expression in the Coral Favia fragum over Diel and Lunar Reproductive Cycles
Source: PLoS One. 2011 May 6;6(5):e19755. doi: 10.1371/journal.pone.0019755 (PMC3089635; doi:10.1371/journal.pone.0019755)
Supplement: Table S2 — Degenerate primer sets. Degenerate primers were based on alignments using A. millepora and N. vectensis sequences using Sequencher software. Primers were selected using PrimerExpress software. (DOCX) [file pone.0019755.s002.docx]

| **Gene** | **Forward Primer (5’- 3’)** | **Reverse Primer (5’ - 3’)** | **Expected length (bp)** |
| --- | --- | --- | --- |
| ***ef1α*** | TGATTGTCCTCMACCATCCAGGTGA | TCCACYTCCTTAATRACRCCAACAGC | 210 |
| ***cry1*** | ATGGKGGWGAGACAGAAGC | CAAAYACCTTCATTCCWKCYTCC | 350 |
| ***cry2*** | TGGATWGATGCAATMATGCGACAG | GTGGGAAGGYKTCTTAKTTCKGG | 260 |
| ***clock*** | CCDTTTGARGTKCTRGGAAC | AATCGRAATWTGAAYGARAAGAARCG | 800 |
| ***cycle*** | TGTAATGCCATGTCACGCAAGCTG | GTGTCCTCCYTTGCATYGGAATCTGTA | 800 |
