# Supplementary material for: Circadian Clock Gene Expression in the Coral Favia fragum over Diel and Lunar Reproductive Cycles
Source: PLoS One. 2011 May 6;6(5):e19755. doi: 10.1371/journal.pone.0019755 (PMC3089635; doi:10.1371/journal.pone.0019755)
Supplement: Table S3 — Statistical analyses of August 2009 diel expression data. Groups showing statistically significant (p<0.05) differences in gene expression as determined using a Kruskal-Wallis one-way analysis on ranks with a Dunn's method post-hoc. (DOCX) [file pone.0019755.s003.docx]

| Gene | Local Time (24h) | Group |
| --- | --- | --- |
| *cry1* | 0200 | C |
|  | 0600 | BC |
|  | 1000 | A |
|  | 1400 | AB |
|  | 1800 | ABC |
|  | 2200 | C |
| *cry2* | 0200 | BC |
|  | 0600 | C |
|  | 1000 | C |
|  | 1400 | AB |
|  | 1800 | A |
|  | 2200 | ABC |
| *clock* | 0200 | ABC |
|  | 0600 | BC |
|  | 1000 | C |
|  | 1400 | AB |
|  | 1800 | A |
|  | 2200 | ABC |
| *cycle* | 0200 | B |
|  | 0600 | B |
|  | 1000 | B |
|  | 1400 | B |
|  | 1800 | AB |
|  | 2200 | A |
